# Testing decision-making competency of schizophrenia participants in clinical trials. A meta-analysis and meta-regression

**DOI:** 10.1186/s12888-017-1580-z

**Published:** 2018-01-05

**Authors:** Sorin Hostiuc, Mugurel Constantin Rusu, Ionut Negoi, Eduard Drima

**Affiliations:** 1 Carol Davila University of Medicine and Pharmacy, Sos.Vitan Barzesti 9, 042122 Sector 4, Bucuresti, Romania; 2 National Institute of Legal Medicine, Bucharest, Romania; 3 University of Medicine and Pharmacy, Galați, Romania

**Keywords:** Informed consent, Schizophrenia, MacCAT-CR

## Abstract

**Background:**

The process of assessing the decision-making capacity of potential subjects before their inclusion in clinical trials is a legal requirement and a moral obligation, as it is essential for respecting their autonomy. This issue is especially important in psychiatry patients (such as those diagnosed with schizophrenia). The primary purpose of this article was to evaluate the degree of impairment in each dimension of decision-making capacity in schizophrenia patients compared to non-mentally-ill controls, as quantified by the (MacCAT-CR) instrument. Secondary objectives were (1) to see whether enhanced consent forms are associated with a significant increase in decision-making capacity in schizophrenia patients, and (2) if decision-making capacity in schizophrenia subjects is dependent on the age, gender, or the inpatient status of the subjects.

**Methods:**

We systematically reviewed the results obtained from three databases: ISI Web of Science, Pubmed, Scopus. Each database was scrutinised using the following keywords: “MacCAT-CR + schizophrenia”, “decision-making capacity + schizophrenia”, and “informed consent + schizophrenia.”

**Results:**

We included 13 studies in the analysis. The effect size between the schizophrenia and the control group was significant, with a difference in means of −4.43 (−5.76; −3.1, *p* < 0.001) for understanding, −1.17 (−1.49, −0.84, p < 0.001) for appreciation, −1.29 (−1.79, −0.79, *p* < 0.001) for reasoning, and −0.05 (−0.9, −0.01, *p* = 0.022) for expressing a choice.

**Conclusions:**

Even if schizophrenia patients have a significantly decreased decision-making capacity compared to non-mentally-ill controls, they should be considered as competent unless very severe changes are identifiable during clinical examination. Enhanced informed consent forms decrease the differences between schizophrenia patients and non-mentally-ill controls (except for the reasoning dimension) and should be used whenever the investigators want to include more ill patients in their clinical trials. Increased age, men gender and an increased percentage of inpatients might increase the differential of decision-making incompetence compared to non-mentally-ill subjects in various dimensions of the decision-making competence as analysed by the MacCAT-CR scale, but the small number of subjects did not allow us (except for one instance) to reach statistical significance.

## Background

Schizophrenia patients, as a group, are considered less able to give a proper consent for either specific medical procedures, or for their inclusion in clinical trials, because they tend to have a decreased capacity to understand, retain and process relevant information [1]. However, various studies suggested that many patients with this illness, even in the acute phase, have an intellectual capacity averaging the one found in the general population. Moreover, once the treatment begins, many deficits are significantly diminished; therefore, some authors consider that the decision making capacity analysis should be mandatory and done multiple times during the course of the treatment [2]. In average, Jeste found that between 10 and 52% of schizophrenia patients do not have insight [3]. The absence of decision-making capacity (DMC) is more important in hospitalized patients [3], if they are in a psychotic state, or if they have a decreased educational level [4].

The process of assessing the DMC of potential subjects before their inclusion in clinical research is a legal requirement and a moral obligation, being essential for respecting their autonomy. The degree of impairment that can render a subject incompetent to participate in a clinical trial should, ideally, reflect a societal judgement about reaching a proper equilibrium between its autonomy and protection as a vulnerable subject [5].

DMC is a four-dimensional concept, which includes (1) the understanding of the disclosed information, (2) its appreciation of a particular setting, (3) the reasoning associated with that information and (4) the aptitude to express a choice [6]. DMC is highly dependent upon the capacity of an individual to choose a specific course of action voluntarily [1, 6, 7] (including the participation to a clinical trial). The choice whether to enter a clinical trial should be fully autonomous. Full autonomy is presumed in adults, unless there are circumstances that can cause a partial or total loss of their capacity to act autonomously (the presence of dementia, schizophrenia or other psychiatric disorders, prisoners, persons with a low socioeconomic status, and so on) [8–10].

Schizophrenia patients have quantitative and qualitative mental deficits, classified in three main categories: cognitive, negative (associated with the disruption of normal behaviours and emotions) and positive (psychotic) symptoms. Their presence can potentially lead to an incorrect assessment of a request to participate in a clinical trial [11]. Howe et al., for example, found that DMC is inversely correlated with specific positive symptom factor scores, especially those associated with cognition (poor attention, difficulty in abstract thinking and conceptual disorientation) [12]. Moser et al. found that patients with positive symptoms do not have significant impairments on any MacArthur Competence Assessment Tool for Clinical Research (MacCAT-CR) subscale, while patients with negative and disorganised symptoms have a decreased DMC [13]. Overall, a larger proportion of people with schizophrenia has a significant degree of decision-making impairment compared to non-mentally-ill subjects [3]. Some studies suggest that various factors such as age, gender or impatient status could alter DMC [9, 14, 15].

The MacCAT-CR instrument is often used to assess the DMC for taking part in clinical research of schizophrenia subjects. It is a 20- to 30-min semi-structured interview, which analyses the four main dimensions of decision-making capacity, each of them receiving a particular score – 0-26 points for understanding, 0–6 points for appreciation, 0–8 points for reasoning and 0–2 points for expressing a choice [16].

Most studies that compared DMC of schizophrenia subjects versus non-mentally-ill controls, by using the MacCAT-CR instrument, found significant decreases in one or more of its main dimensions [13, 15, 17–22]. The mean score for each dimension and the score difference between the subgroups was variable. A meta-analysis performed by Wang et al. analysed the decisional capacity measured by MacCAT tools in schizophrenia patients; however, it failed to include many studies, and it did not test specifically decisional capacity for participating in clinical trials [23]. Enhanced informed consent forms are often use to increase the capacity of the patients to understand relevant information about their medical condition and the proposed medical interventions [24–26], including schizophrenia patients [25].

The *primary aim* of this article was to test the impairment in each dimension of DMC in schizophrenia patients compared to non-psychiatric controls, as appraised by the MacCAT-CR instrument. *Secondary objectives* were (1) to see whether enhanced informed consent (EIC) is associated with a significant increase in DMC in schizophrenia patients, and (2) if the DMC in schizophrenia subjects depends on the age, gender, their inpatient status, or country of origin.

## Methods

We performed the study according to the PRISMA and MOOSE guidelines for reporting systematic reviews and meta-analyses of observational studies in epidemiology.

### Selection criteria

Inclusion criteria: (1) observational studies that analysed the DMC of schizophrenia subjects compared to a control group comprising of non-mentally-ill subjects, with the aid of the MacCAT-CR instrument. The main exclusion criteria were: (1) not fulfilling the inclusion criteria, (2) not presenting enough information to reconstruct the data needed for the analysis, (3) case-series studies and studies without a control group.

### Search method.

We analysed the results found in three databases: ISI Web of Science, Pubmed, Scopus. For each, we used the following keywords: “MacCAT-CR + schizophrenia”, “decision-making capacity + schizophrenia”, and “informed consent + schizophrenia”,

### Data collection and analysis

Two authors researched the databases independently, with an agreement rate for the added articles of 83.3%. Data was then extracted separately by the two reviewers and listed in an Excel file. When we found discrepancies between the obtained results, the issues were re-analyzed by both examiners. We summarized the following data: study, year, total number of cases, mean age and standard deviation, gender ratio for each group, ratio of in- versus outpatients in the cases group, and mean and standard deviation of the values in the four main dimensions of MacCAT-CR - understanding, appreciation, reasoning and expressing a choice. If a particular article contained data regarding a potential intervention aimed at improving the DMC, we added the data for both unenhanced and enhanced informed consent techniques/forms.

The risk of bias was assessed separately for each case.

The quality assessment was performed by two researchers using the Newcastle-Ottawa Scale for case-control studies (NOS) [27]. NOS was developed to address the quality of non-randomized studies for systematic reviews/meta-analyses. Briefly, it comprises of eight items, categorised into three main groups: (1) selection of the survey groups, (2) comparability of the two groups and (3) evaluation of the exposure or outcome of interest. The final quality score was computed as an average of the scores given by the two reviewers. The agreement rate between reviewers was 90%.

### Statistical analysis

We determined the effect size as the difference in means in all cases using a random effects model computed in Comprehensive Meta-analysis software. To assess the odds for significant alterations of the dimensions of the DMC, we calculated the Odds Ratio (OR). This was done by transforming the difference in means in Cohen’s d (by dividing it by the standard deviation), converting Cohen’s d into ln(OR) through the following formula ln(OR) = dπ/sqrt(3), and then ln(OR) to OR through the following eq. OR = e^ln(OR)^. For each group and subgroup, we performed a forest plot. We used Egger’s intercept technique for the analysis of publication bias, and I^2^ for measuring the heterogeneity. For comparison of the effect size between two groups, we used the Z-test. We used 95% confidence intervals and considered a *p-value* of 0.05 or lower to be statistically significant. We rounded the obtained values to the second decimal, except for (1) *p*-values below 0.05, case in which we included data until the 4th decimal, (2) when by rounding, the value was below 0.01 and (3) when by rounding the OR, we obtained the value 1.

## Results

### Search synthesis

**Fig. 1:**
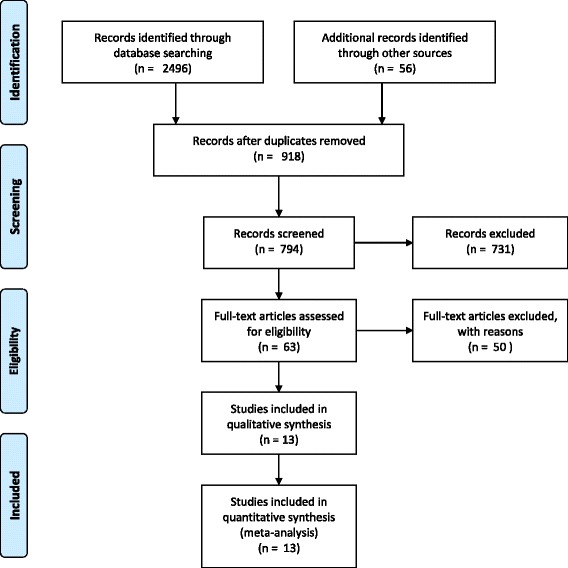
Selection algorithm for the included studies (PRISMA flow diagram)

We obtained 2496 results from which, after deleting duplicate and irrelevant studies and analysing the type of paper and abstracts (if available), 56 articles were selected. They were downloaded and examined further. Evaluating the references of these 53 articles, we identified seven more that were considered potentially relevant, which were also downloaded. From the total number of 60 studies, we selected 13, which ultimately fulfilled the inclusion criteria, and were added to the meta-analysis (with the except of the “expressing a choice” subscale, where there were only 10 studies with relevant information). We excluded 47 articles for not meeting the inclusion criteria. Details regarding the selection algorithm are shown in Fig. 1. The studies included in the analysis are described in detail in Table 1.

**Table 1:** Summary of studies included in the meta-analysis

Study	Total No Subjects	Schiz. Subjects	Control group	Mean age (St dev)^a^	Men%^a^	Description	Quality^c^
2008, Candillis [18]	109	52	57	37.79(11.67), 41.04(13.16)	76.9, 57.9	Subjects: 45 stable patients from a state hospital, seven outpatients. Controls: patients from a diabetes clinic Answers were given regarding the participation to a potential drug trial; payment of 10$ per participation	5
2000, Carpenter [19]	54	30	24	40.2(8.8), 39.7(10.2)	56.7, 78.2	Subjects: inpatients and outpatients (20 and 10 respectively). 28 schizophrenia patients, 2 patients with schizoaffective disorder. Controls: recruited from community centres and a free medical clinic. Answers regarding the participation to a randomised, double-blind trial for a novel anti-psychotic medication	5
2004, Cohen [34]	26	6	20	40.0 (7.8), 41.1 (10.3)	33.3, 60	Subjects: 6 inpatients. Controls: community volunteers Answers: regarding two studies – a drug study, and a ketamine study. We averaged the values of the two studies for each MacCAT CR subscale	5
2012, Harmell [20]	17	9	8	57(10), 52.2(12.1)	89, 50	Subjects: outpatients, recruited through a registry; randomly assigned for receiving either a normal or a web-media enhanced consent procedure. Controls: non-psychiatry patients, recruited through a registry Answers: regarding a hypothetical clinical trial for an experimental cognition enhancing medication	6.5
*2012, Harmell* [20]	*18*	*10*	*8*	*57.9(8.9), 48.6(15.9)*	*40, 62.5*		
2009, Jeste [21]	95	66	29	51.2(6.5), 54.2(9.3)	64, 52	Subjects: community-dwelling outpatients aged >40 years, with schizophrenia. Subjects were randomly assigned to either a standard or a multimedia consent procedure. Controls: recruited through newspaper advertisements, flyers, or word of mouth Answers: regarding a 14-week double-blind, placebo-controlled RCT to determine the effectiveness of a cognition-enhancing drug for cognitive deficits associated with schizophrenia or with normal ageing	5
*2009, Jeste* [21]	*93*	*62*	*31*	*52.4(8), 54.7(7.3)*	*65, 45*		
2007, Kim [22]	131	91	40	42.2(10.2), 39.9(10.9)		Subjects: with severe mental illness consisted of two subgroups: 55 participants from a schizophrenia study from six different sites across the US; 36 people from two outpatient clinics serving individuals with severe and persistent mental illnesses, and from inpatient units. Controls: recruited through advertisements from the community, in support staff work areas of a general hospital and at an out-patient substance misuse recovery program Answers: regarding participation in the CATIE study	6
2003, Kovnick [15]	51	27	24	39.1(7.1), 39.7(10.2)	78, 79	Cases: 27 psychiatric inpatients, non-acutely ill. Controls: individuals from the community, without known psychiatric pathologies Answers regarding a hypothetical randomized, double blind trial for a new schizophrenia drug	5
2016, López-Jaramillo [35]	120	80	40	34.9(10.5), 37(14.3)	73,47	Cases: 80 individuals with schizophrenia. Controls: healthy volunteers Answers regarding the participation to a clinical trial	6
2006, Moser [36]	60	30	30	34.1(10.65), 30(11.46)	73, 87	Cases: 30 individuals with schizophrenia (6 outpatients, 24 inpatients). Controls: healthy individuals, without significant psychiatric or medical pathologies Answers regarding a possible double-blind, placebo-controlled trial of a cognitive-enhancing agent called Synaptoclear	4.5
*2006, Moser* [36]	*60*	*30*	*30*	*34.1(10.65), 30(11.46)*	*73, 87*		
2002, Moser [13]	50	25	25	31.56(9.77), 37.4(7.76)	84, 76	Cases: 25 individuals with schizophrenia, 21 outpatients, and four inpatients, 18 of which received antipsychotic medication. Controls: 25 infected individuals, 24 outpatients, one inpatient, 15 under psychotropic medication (primarily for depression); none was under antipsychotic medication Answers regarding a hypothetical 6-week, randomised, double-blind, placebo-con- trolled study of a cognition-enhancing agent called Synaptoclear	5.5
2005, Palmer [33]	71	35	36	65.7(5.2), 70.9(6.2)	57.1, 97.2	Cases: 35 clinically stable outpatients with diagnoses of schizophrenia (30) or schizoaffective disorder (5). Controls: 36 outpatients with diabetes mellitus, recruited through clinical research programs Answers regarding participation in a randomised controlled trial for an experimental compound (“plakmin”), tested for cognitive-enhancing effects, which was modelled after a local study that tested the cognitive benefits of a cholinomimetic agent	5
2007, Palmer [37]	59	31	28	52.4(7), 56.6(11.1)	48.4,46.4	Cases: 31 outpatients with schizophrenia. Controls: recruited from the community (28) Answers regarding a longitudinal study of side effects, including tardive dyskinesia, of FDA–approved second-generation antipsychotic medications among middle-aged and older patients	5.5
2015, Wang [38]	128	100	28	35.85(11.21), 45.68(11.54)	56, 53	Cases: both inpatients and outpatients. Controls: community volunteers Answers regarding the participation to a hypothetical clinical trial	4.5

^a^statistics for both schizophrenia patients and controls, separated by a comma

^b^italic lines – decision-making capacity after using enhancement techniques

^c^Newcastle-Ottawa Scale for case-control studies

### Quality assessment

The studies had quality scores ranging from 4.5 to 6.5 using the NOS Case Control scale. The values for each study are presented in Table 1.

### Descriptive data

Within the 13 included studies were 684 schizophrenia subjects (582 in standard consent studies and 102 in enhanced consent studies), and 458 subjects in the control groups (389 in standard consent studies and 69 in enhanced consent studies). The arithmetic mean values for the four dimensions of the MacCAT-CR test are presented in Table 2. Eleven studies were performed in the US and two outside US (one in China, and one in Colombia).

**Table 2:** Mean scores for the included studies computed as simple arithmetic means

Parameter	Standard Informed Consent, Cases	Standard Informed Consent, Controls	Mean values	Enhanced Informed Consent, Cases	Enhanced Informed Consent, Controls
Understanding	17.8	23.7	20.16	22.29	25.13
Appreciation	3.98	5.36	4.53	5.03	5.61
Reasoning	4.96	6.18	5.45	5.46	5.63
Expressing a choice	1.90	1.97	1.93	1.99	1.99
Total No. of Subjects	582	389		102	69

### Understanding

**Fig. 2:**
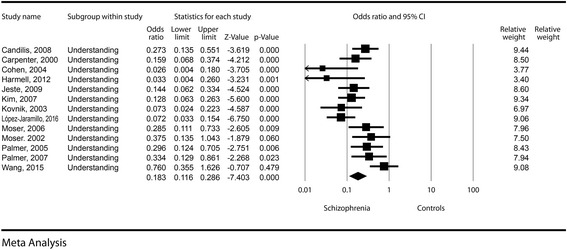
Understanding – Forest plot for ORs

The effect size between the schizophrenia subjects and the control group was significant, with a difference in means of −4.43 (−5.76; −3.1), *p* < 0.001. The odds for a decreased understanding in schizophrenia patients was about five times higher compared to control groups (OR = 0.18, CI = 0.12–0.29, *p* < 0.001). See Fig. 2 for details. Publication bias was not statistically significant (Egger’s regression intercept = −2.6, *p* = 0.17). The heterogeneity of the understanding dimension was low (I^2^ = 9.84%).

By running a meta-regression using mean age, percent of men and percent of inpatient status as covariates, we could not find any statistically significant effects (see Table 3). The difference between mean values for understanding in cases versus control groups was larger for studies conducted outside the US (−4.67, with limits between −12.16 and 2.836) compared to studies performed in the US (−3.941, with limits between −5.02 and −2.86). The difference was statistically significant (Z = −7.26, *p* < 0.001).

**Table 3:** Meta regression analysis

	Percent Men (B, Z)	Mean age in the schizophrenia group (B, Z)	Inpatient status (B, Z)
Understanding	−0.02, −1.56	−0.01, −0.56	−0.002, −0.33
Appreciation	−0.007, −0.79	−0.002, −0.12	−0.005, −1.15
Reasoning	*−0.02, −2.54*	0.01, 0.43	−0.002, −0.29
Expressing a choice	−0.004, −0.55	0.008, 0.58	−0.000, −0.11

*B* Beta coefficient, *Z* Z-value, Italicized cells – statistically significant results

Three studies contained information about enhanced informed consent. In them, we found a decreased difference in means compared to controls (−2.73, with limits between −4.97 and −0.49). The odds for a decreased understanding in schizophrenia subjects using EIC was almost four times higher compared to the control groups (OR = 0.28, CI = 0.14–0.59, *p* = 0.001).

The use of EIC leads to a significant increase in understanding in schizophrenia subjects compared to standard informed consent forms (Z = −13.34, *p* < 0.001).

### Appreciation

**Fig. 3:**
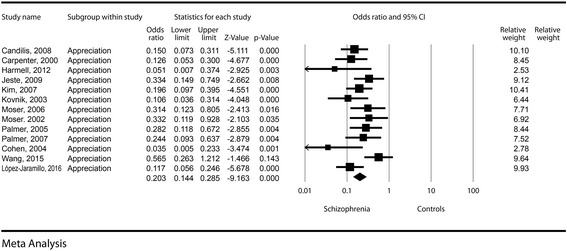
Appreciation – Forest plot for ORs

The effect size was significant, with a difference in means of −1.17 (−1.49, −0.84, *p* < 0.001). The odds for a decreased appreciation in schizophrenia patients was about five times higher compared to the control groups (OR = 0.20, CI = 0.14–0.28, *p* < 0.001). See Fig. 3 for details. Publication bias was not statistically significant (Egger’s regression intercept =1.51, *p* = 0.16). The heterogeneity of the appreciation dimension was very low (I^2^ = 5.79%).

By running a meta-regression using mean age, percent of men and percent of inpatient status as covariates, we could not find any statistically significant effects (see Table 3). The difference between mean values for appreciation in the cases versus control groups was larger for studies conducted outside the US (−1.15, with limits between −2.23 and −0.08) compared to studies performed in the US (−1.01, with limits between −1.31 and −0.72). The difference was statistically significant (Z = −7.021, *p* < 0.001).

Three studies contained information about EIC. In them, we found a decreased difference in means compared to controls (−0.46, with limits between −0.76 and −0.15).

The use of EIC leads to a significant increase in appreciation in schizophrenia subjects compared to standard informed consent forms (Z = −12.525, *p* < 0.001).

### Reasoning

**Fig. 4:**
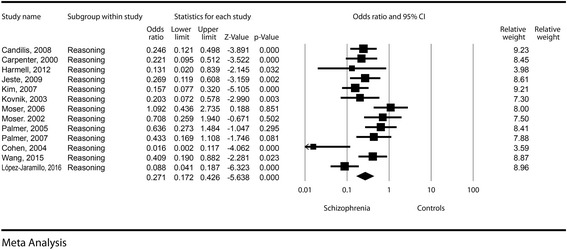
Reasoning – Forest plot for ORs

The effect size was highly significant, with a difference in means of −1.29 (−1.79, −0.79, *p* < 0.001). The odds for a decreased reasoning in schizophrenia patients was almost four times higher compared to the control groups (OR = 0.27, CI = 0.17–0.42, *p* < 0.001). See Fig. 4 for details. Publication bias was not statistically significant (Egger’s regression intercept = 1.73, *p* = 0.11). The heterogeneity of the reasoning dimension was small (I^2^ = 10.8%).

**Fig. 5:**
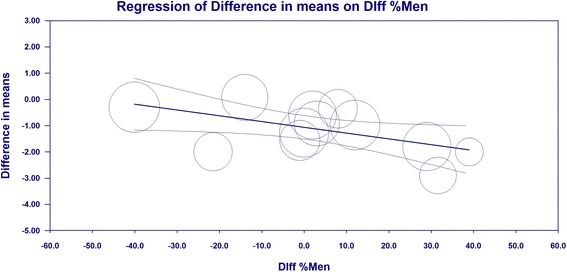
Reasoning – meta-regression, age of schizophrenia subjects

An increase in the percentage of men in the schizophrenia group caused a statistically significant decrease in the difference in means between the cases and control groups (see Table 3 and Fig. 5). The difference between mean values for reasoning in the cases and control groups was larger for studies conducted outside the US (−2, with limits between −4.14 and 0.14) compared to studies performed in the US (−1.09, with limits between −1.53 and −0.64). The difference was statistically significant (Z = −7.021, *p* < 0.001).

Three studies contained information about EIC. In them, we found a non-statistically significant decrease in the difference in means compared to controls (−0.830, with limits between −2.28 and 0.57). The odds for a decreased reasoning in schizophrenia subjects using EIC was four times higher compared to the control groups (OR = 0.26, CI = 0.03–2.60, *p* = 0.250), but the result was not statistically significant.

The use of EIC leads to a significant increase in understanding in schizophrenia subjects compared to standard informed consent forms (Z = −5.05, *p* < 0.001).

### Expressing a choice

**Fig. 6:**
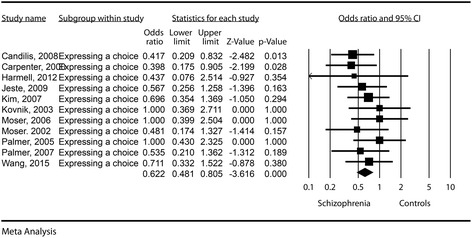
Expressing a choice – Forest plot for ORs

The effect size was significant, with a difference in means of −0.05 (−0.9, −0.01, *p* = 0.022). See Fig. 6 for details. The odds for a decreased aptitude to express a choice in schizophrenia patients was about 66% higher compared to the control groups (OR = 0.62, CI = 0.48–0.80, *p* < 0.001). Publication bias was not statistically significant (Egger’s regression intercept = 0.14, *p* = 0.89). The heterogeneity of the aptitude to express a choice was very low (I^2^ = 0).

By running a meta-regression using mean age, percent of men and percent of inpatient status we could not find any statistically significant differences (see Table 3).

Three studies contained information about EIC. In them, we found a non-statistically significant increase in the difference in means compared to controls (0.01, with limits between −0.03 and 0.5). The odds for a decreased capacity to express a choice in schizophrenia subjects using the EIC was similar to the one found in the control groups (OR = 1.09, CI = 0.62–1.91, *p* = 0.76).

The use of EIC forms leads to a significant increase in the aptitude to express a choice compared to standard informed consent forms (Z = −3.163, *p* = 0.002).

## Discussions

Our study showed that, when using non-enhanced informed consent procedures, schizophrenia subjects tend to have significantly decreased values for all dimensions of DMC, the highest effect sizes being encountered for the understanding and reasoning sub-scales.

Psychiatry patients are considered a vulnerable population in clinical research, and their inclusion in a trial can be done only in particular circumstances. For example, the CIOMS guidelines state that research involving individuals who, by reason of mental or behavioral disorders are not capable of giving adequately informed consent, can only be done if there are no other subjects that could be equally involved in that particular trial, and who are able to give an adequately informed consent [28]. Therefore, to include subjects in clinical trials, the investigators must make a binary decision about the presence/absence of DMC. Some studies recommend various threshold values for some or all DMC parameters [22, 29]. For example, in the Clinical Antipsychotic Trials of Intervention Effectiveness – Schizophrenia (CATIE) study [30], the investigators established a threshold value of 15 for the understanding scale, a value that was proven to be a little too conservative [22]. Our study showed that there is a circa 4-point difference between schizophrenia and control subjects in the understanding scale. If we were to take into account the mean value for understanding (20.16, see Table 2) and add to this the average difference obtained in the meta-analysis (−4.43), we would see that the mean theorised value for the schizophrenia subjects [20.16-(4.43/2) = 17.95] is well above this threshold. Similarly, the lower limit with a 95%CI (−5.76, corresponding to a lower limit for the schizophrenia group of 18.06) is well above the 15 points threshold. By also considering the results of Kim et al. [22], our analysis emphasizes the idea that schizophrenia patients should be considered, per prima facie, as being able to make informed decisions regarding their participation to clinical trials. By assuming decision-making incapacity in these patients, we might discriminate them based on their disease; therefore, by trying to obey the bioethical principle of autonomy, we might breach the principle of justice.

Jeste et al. suggested the presence of a high inter-group heterogeneity in DMC for patients with schizophrenia, with a standard deviation often increased twofold compared to the control groups, and hinted as one of its leading sources the variable inclusion of in- and outpatients in the cases groups [3]. Our analysis failed to confirm his hypothesis, as all four dimensions of decision-making capacity were insignificantly affected by the percentage of inpatients included in the initial studies. Also, our analysis showed a little heterogeneity between studies, suggesting an excellent reliability of the results.

The scores on every subscale decreased once the percentage of men in the schizophrenia group increased, but the result did failed to reach statistical significance (except for the reasoning subscale). This failure could be generated either by a low number of studies included in the analysis, or an actual absence of an association between gender and decision capacity. Some studies published in the scientific literature showed that women have a better social adaptability to the disease. Hintikka et al. found that women with schizophrenia have significantly better independent skills and domestic activities compared to men with the same illness. For example, 11% of men lacked skills regarding personal hygiene compared to only 4% of the women; 32% of men lacked skills regarding financial affairs compared to 20% in women; 25% of men lacked decision-making capacity compared to only 19% in women [14]. Hambrecht et al. showed that maladaptive social behaviours (including negative symptoms or inappropriate illness behaviours) were more often found in men with schizophrenia [31]. Palmer and Jeste revealed that understanding is correlated with the severity of negative symptoms [32]. However, more studies are needed before we can definitely associate (or fail to associate) gender with decreased decision capacity in schizophrenia patients.

Various studies have suggested that age could alter decision-making capacity in schizophrenia patients (see e.g. [32]). Our study showed that there might be an age-related deterioration in various MacCAT-CR subscales, but we could not prove it with statistical significance. This failure could be generated either by a low number of studies included in the analysis, or an actual absence of an association between age and decision capacity.

Using EICs, various authors proved a significant increase in DMC [13, 20, 21]. Our study shows that, compared to the control group, EICs decreased the deficits in the understanding, reasoning and appreciation subscales. When compared to standard consent studies, EICs significantly increased the values for all four MacCAT-CR subscales. Based on these results, we recommend using EICs especially in clinical trials that require subjects with severe cognitive deficits.

Kim et al. found that understanding sub-scale from MacCAT-CR was more important as a predictor of a categorical capacity status compared to appreciation or reasoning [22]. Most instruments used for assessing the decision-making capacity only test the understanding (for details see Palmer et al. [33]). Our study shows that reasoning, together with understanding are the most affected dimensions of decision-making capacity in schizophrenia subjects, and therefore, tasks directed specifically toward increasing them might be the best approach in optimising the decision-making capacity for potential subjects with schizophrenia and decision-making incapacity. Additionally, if researchers would like to simplify DMC analysis, reasoning should be tested alongside understanding to improve the accuracy of the outcome.

The results of this article could be used by clinical researchers to better fine-tune the selection criteria for inclusion in clinical trials, and by the Institutional Ethics Committees to verify the compliance of the study protocol with the general and specific ethical principles of clinical research.

### Limits

The number of studies included in the analysis is small (13); however, if we were to include studies in which decision-making capacity was evaluated using other scales, the results would have been more heterogeneous, mainly due to the usage of distinct methodologies for assessing DMC. Moreover, only three studies included data about enhanced ways of informing potential subjects. Even if the number was small, the results reached statistical significance in most scales, suggesting that they profoundly improved DMC. Only two studies were performed outside US, potentially making the results geographically biased. Therefore, they should be interpreted with caution in significantly different population groups. Also, the comparisons US-outside US should be regarded as potentially having poor error estimation.

## Conclusions

Even if schizophrenia patients have a significantly decreased decision-making capacity compared to non-mentally-ill controls, they should be considered as competent unless very severe changes are identifiable during clinical examination. Enhanced informed consent forms decrease the differences between schizophrenia patients and non-mentally-ill controls (except for the reasoning dimension) and should be used whenever the investigators want to include more ill patients in their clinical trials. Increased age, men gender and an increased percentage of inpatients might increase the differential of decision-making incompetence compared to non-mentally-ill subjects in various dimensions of the decision-making competence as analysed by the MacCAT-CR scale, but the small number of subjects did not allow us (except for one instance) to reach statistical significance.

## Abbreviations

CATIE: Clinical Antipsychotic Trials of Intervention Effectiveness – Schizophrenia
CIOMS: Council for International Organizations of Medical Sciences
DMC: Decision making capacity
EIC: Enhanced informed consent
MacCAT-CR: MacArthur Competence Assessment Tool for Clinical Research
NOS: Newcastle-Ottawa scale
OR: Odds Ratio
